# Modulating Nrf2 to control lipid peroxidation and ferroptosis: implications for cancer management

**DOI:** 10.3389/fonc.2025.1701249

**Published:** 2025-12-03

**Authors:** Ramazan Tunc, Ilker Ates, Beyza Yılmaz, Alessandro Medoro, Sergio Davinelli, Sibel Suzen, Luciano Saso

**Affiliations:** 1 Department of Pharmaceutical Chemistry, Faculty of Pharmacy, Ankara University, Ankara, Türkiye; 2 Graduate School of Health Sciences, Ankara University, Ankara, Türkiye; 3 Department of Pharmaceutical Toxicology, Faculty of Pharmacy, Ankara University, Ankara, Türkiye; 4 Department of Medicine and Health Sciences “V. Tiberio”, University of Molise, Campobasso, Italy; 5 Department of Physiology and Pharmacology ‘‘Vittorio Erspamer”, Sapienza University of Rome, Rome, Italy

**Keywords:** Nrf2, Nrf2 modulators, lipid peroxidation, ferroptosis, oxidative stress, cancer

## Abstract

Nuclear factor erythroid 2–related factor 2 (Nrf2) is a key transcription factor that regulates the expression of genes involved in cellular protection against oxidative stress and the maintenance of redox homeostasis. In addition to modulating reactive oxygen species (ROS), Nrf2 influences lipid peroxidation (LP) and ferroptosis, an iron-dependent form of regulated cell death driven by lipid peroxide accumulation. Dysregulation of Nrf2 signaling has been implicated in the pathogenesis and progression of several malignancies, where it affects tumor cell survival, chemoresistance, and metabolic reprogramming. This review summarizes current knowledge on the complex relationship between Nrf2 activity, LP dynamics, and ferroptotic mechanisms in cancer biology. It also highlights how Nrf2-dependent transcriptional programs not only regulate antioxidant responses but also contribute to cellular detoxification, metabolism, autophagy, and proteostasis, processes closely linked to tumorigenesis and cancer cell adaptability. Given that Nrf2 may have a dual role in cancer, either promoting cytoprotection or supporting tumor progression and chemoresistance, we discuss emerging strategies to selectively modulate Nrf2 and ferroptosis-related pathways to increase cancer cell sensitivity to oxidative damage and reduce resistance.

## Introduction

Lipid peroxidation (LP) represents a fundamental mechanism of oxidative damage of fatty acids, typically polyunsaturated fatty acids (PUFAs), in cellular membranes and occurs when reactive oxygen species (ROS) extract a hydrogen atom from bis-allylic methylene groups, generating lipid radicals. These radicals react with molecular oxygen to form lipid peroxyl radicals, which in turn propagate the chain reaction by attacking adjacent lipids. The process culminates in the accumulation of lipid hydroperoxides and reactive aldehydes, such as malondialdehyde (MDA) and 4-hydroxynonenal (4-HNE), which disrupt membrane architecture, impair protein function, and trigger cell death pathways (1).

Ferroptosis is critically dependent on this process. Unlike other forms of cell death, such as apoptosis or necrosis, ferroptosis is defined by the accumulation of lipid peroxides beyond the detoxification capacity of the cell. The failure of antioxidant systems, particularly glutathione peroxidase 4 (GPX4), which reduces lipid hydroperoxides to non-toxic lipid alcohols, leads to the collapse of membrane integrity and ferroptotic cell death (2, 3). As such, lipid peroxidation is not merely associated with ferroptosis but serves as both a trigger and a modulator, positioning LP as a mechanistic hallmark of this form of cell death. This process is particularly relevant in therapy-resistant tumors, in which ferroptosis activation has shown potential to overcome resistance mechanisms. Furthermore, ferroptosis is involved in the pathogenesis of several degenerative diseases associated with oxidative stress (OS), highlighting its therapeutic relevance (4).

A central regulator of ferroptosis and oxidative stress responses is nuclear factor erythroid 2-related factor 2 (Nrf2), a transcription factor that controls the expression of a wide array of antioxidant and cytoprotective genes. Nrf2 plays a pivotal role in redox homeostasis and is involved in key metabolic processes, including xenobiotic detoxification, iron and heme metabolism, lipid and carbohydrate metabolism, proteostasis, and the regulation of cell death pathways (5).

The activity of Nrf2 is primarily regulated by Kelch-like ECH-associated protein 1 (Keap1), a cytosolic repressor that acts as a substrate adaptor for a Cullin-3-based E3 ubiquitin ligase complex. Under basal conditions, Keap1 binds Nrf2 and promotes its proteasomal degradation, thereby limiting its transcriptional activity. Upon exposure to oxidative or electrophilic stress, critical cysteine residues on Keap1 are modified, resulting in the stabilization and nuclear accumulation of Nrf2. Once in the nucleus, Nrf2 binds to antioxidant response elements (AREs) and induces the expression of target genes involved in glutathione synthesis, iron sequestration, and detoxification of lipid peroxides (6).

In the cancer context, aberrant activation of the Nrf2 is frequently observed and contributes to tumor progression by counteracting lipid peroxidation and ferroptosis. Many tumor types, including lung, liver, and head and neck carcinomas, harbor mutations in *KEAP1* or *NRF2* that lead to sustained Nrf2 activity. This persistent activation enhances the antioxidant and iron-sequestering capacity of cancer cells, promoting glutathione synthesis, increasing the expression of ferroptosis-suppressor proteins, and reducing free iron availability (7). As a result, these cells acquire a metabolic advantage that allows them to resist high levels of ROS and evade ferroptotic cell death. While this mechanism protects normal cells from oxidative damage under stress, in cancer, it facilitates proliferation, chemoresistance, and immune evasion. However, Nrf2 does not play a uniformly oncogenic role. In the early stages of oncogenesis, transient activation of Nrf2 can exert tumor-suppressive effects by enhancing the antioxidant response, limiting genomic instability, and promoting the elimination of transformed or damaged cells. This suggests a context-dependent function, where Nrf2 initially acts as a guardian against malignant transformation but may later be co-opted by cancer cells to support their survival and resistance. This duality poses both challenges and opportunities: inhibiting Nrf2 may sensitize cancer cells to ferroptosis inducers, whereas activating Nrf2 may offer protection in non-neoplastic diseases driven by ferroptosis and oxidative stress (8).

## Lipid peroxidation and its role in ferroptosis

Lipid peroxidation (LP) is a defining feature of oxidative damage and a central mediator of ferroptosis. It involves the oxidative degradation of polyunsaturated fatty acids (PUFAs) within cellular membranes. Triggered by reactive oxygen species (ROS), the process begins with hydrogen abstraction from bis-allylic methylene groups, producing lipid radicals that react with molecular oxygen to form lipid peroxyl radicals. These radicals propagate the chain reaction, leading to the accumulation of lipid hydroperoxides (LOOHs) and reactive aldehydes such as malondialdehyde (MDA) and 4-hydroxynonenal (4-HNE). These byproducts destabilize membranes, disrupt protein function, and activate pro-death signaling. Mitochondrial and plasma membranes, enriched in PUFAs, are particularly susceptible under conditions of iron overload and impaired antioxidant defense (9).

LP is tightly linked to the cellular redox state. Alongside lipid radicals, cells under oxidative stress generate diverse ROS and reactive nitrogen species (RNS) capable of damaging lipids, proteins, and nucleic acids. Chronic oxidative stress, driven by metabolic or environmental factors, amplifies LP and activates regulated cell death pathways, including ferroptosis (10–12). Despite their high reactivity, ROS and RNS also function as signaling molecules in processes such as proliferation, immunity, inflammation, and autophagy. This dual nature, balancing signaling and damage, remains a subject of intense debate (13–15).

ROS include radical species (O_2_•^-^, •OH, RO•, RO_2_•, ¹O_2_, O_3_) and non-radicals such as hydrogen peroxide (H_2_O_2_). RNS encompass molecules like nitric oxide (•NO), nitrogen dioxide (•NO_2_), and peroxynitrite (ONOO^-^). These species are produced through mitochondrial respiration, particularly the tricarboxylic acid (TCA) cycle, and by enzymes including NADPH oxidase (NOX), xanthine oxidase, cytochrome P450s, and nitric oxide synthase (NOS). The reaction of NO with superoxide anion generates ONOO^-^, a potent oxidant capable of damaging proteins and lipids (16, 17).

The chemical course of LP proceeds through initiation, propagation, and termination. In the initiation phase, hydrogen abstraction generates a lipid radical (L•), which reacts with oxygen to form a lipid peroxyl radical (LOO•). This radical perpetuates the chain reaction by abstracting hydrogen from neighboring lipids. Termination occurs through radical–radical interactions or antioxidant-mediated quenching, forming more stable byproducts while leaving behind oxidative stress residues (18, 19).

LP proceeds via both non-enzymatic and enzymatic routes. Non-enzymatic LP is predominantly iron-driven: Fe²^+^ catalyzes the Fenton reaction (Equation 1), converting hydrogen peroxide into hydroxyl radicals (•OH) that rapidly oxidize PUFAs—central to ferroptosis (19). Enzymatic LP involves cyclooxygenases (COXs), lipoxygenases (LOXs), and cytochrome P450s (CYPs). COXs oxidize arachidonic acid into prostaglandins, modulating inflammation and apoptosis (20). LOXs catalyze PUFA peroxidation and play roles in immune responses (21). Cytochrome P450 oxidoreductase (POR), located on the endoplasmic reticulum, participates in lipid oxidation via CYP activation (22).

(1) $$\text{F}{\text{e}}^{3+}+\;{\text{H}}_{2}{\text{O}}_{2}\to \;\text{F}{\text{e}}^{3+}{\text{H}}_{2}{\text{O}}_{2}$$

(2) $$\text{F}{\text{e}}^{3+}{\text{H}}_{2}{\text{O}}_{2}\to \;\text{F}{\text{e}}^{2+}+\;•\text{H}{\text{O}}_{2}+\;{\text{H}}^{+}$$

(3) $$\text{F}{\text{e}}^{3+}+\;•\text{H}{\text{O}}_{2}\to \;\text{F}{\text{e}}^{2+}+\;{\text{O}}_{2}+\;{\text{H}}^{+}$$

(4) $$\text{F}{\text{e}}^{2+}+\;{\text{H}}_{2}{\text{O}}_{2}\to \;\text{F}{\text{e}}^{3+}+\;•\text{OH}\;+\;\text{O}{\text{H}}^{-}$$

Equation 1: Fenton reaction.

When examining the Fenton reaction (Equations 1–4) *in vitro*, the oxidation processes undergone by free radicals can be monitored using various model studies. One of the most well-known methods is that performed with hydrogen peroxide and iron salts (23). In many publications, EDTA has been selected as a reagent for both inhibiting and accelerating oxidation (24).

*In vivo*, Fe2+ ions have been found in the cell cytosol of organisms. Excessive levels of biogenic iron in the body are known to be associated with the pathology of certain diseases. These include inflammation, neurodegeneration, neuroinflammation, and even cancer (25, 26). Some sources have reported that excess iron in the body may also be associated with the development of liver disorders, cardiovascular diseases, and lung diseases (27, 28). The Fenton reaction is gaining attention in some *in vivo* applications. For example, nanotechnology methods involving polymer nanoparticles and light-sensitive nanoparticles have been used in many successful applications where the Fenton reaction has been adapted to the cancer microenvironment. It has been observed that the Fenton reaction, catalyzed by light-sensitive nanoparticles, leads to healing in cancer cells (29). The OH• radical released by the Fenton reaction is one of the most reactive molecules that causes oxidative damage in cells. This property is considered a potential therapeutic approach to prevent cancer development. The mechanisms of action of some known cancer drugs, such as doxorubicin (DOX) [13], β-lapachone [14], and cisplatin [15], are known to involve attacking tumor cells with the OH• radical via the Fenton reaction (30, 31).

To mitigate LOOH-induced toxicity, cells rely on glutathione peroxidase 4 (GPX4), a selenoenzyme that reduces lipid hydroperoxides to inert alcohols using glutathione (GSH) as an electron donor. Inhibition of GPX4—whether pharmacologically (e.g., by erastin or RSL3) or via GSH depletion—leads to lipid peroxide accumulation, membrane disruption, and ferroptotic death (32).

**Figure 1:**
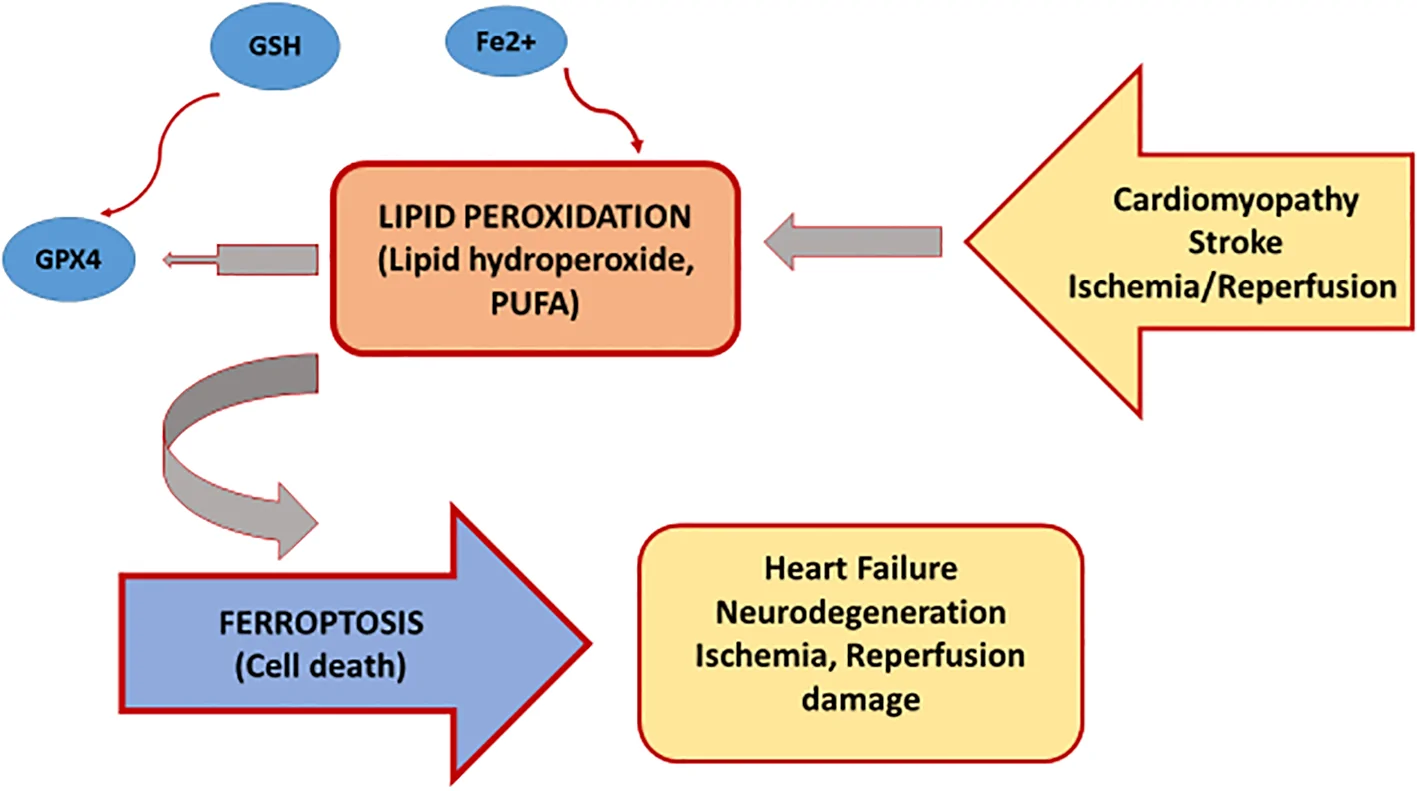
The role of ferroptosis and lipid peroxidation in the development of diseases.

Ferroptosis is defined by three core biochemical events: (i) PUFA-phospholipid oxidation, (ii) the presence of redox-active iron, and (iii) compromised antioxidant defenses, particularly GSH and GPX4 (33, 34) (**Figure 1**). Mitochondrial TCA cycle activity, fatty acid β-oxidation, and amino acid metabolism contribute to ROS generation that, in concert with iron, amplify LP and drive ferroptotic signaling (35, 36).

Morphologically, ferroptosis diverges from classical apoptosis and necrosis. It lacks features such as apoptotic body formation or necrotic lysis. Instead, it is marked by shrunken mitochondria with dense membranes and ruptured outer membranes. Biochemical hallmarks include GSH depletion, GPX4 inactivation, and lipid peroxide accumulation (37–39).

Ferroptosis plays a pathogenic role in ischemia–reperfusion injury (IRI). Upon reperfusion, elevated iron levels exacerbate ROS generation and LP, triggering ferroptotic cell death. This amplifies tissue injury and highlights ferroptosis as a therapeutic target for IRI-related pathologies (40, 41).

In cancer cells, chronic oxidative stress often creates selective vulnerability due to impaired GSH metabolism. Under these conditions, the GPX4–GSH axis becomes critical for survival. When GSH is depleted, GPX4 fails to detoxify lipid peroxides, culminating in ferroptosis. Conversely, tumor cells that upregulate GPX4 or maintain high GSH pools can evade ferroptosis, supporting proliferation and resistance to oxidative therapies (42, 43).

### Nrf2 as a central regulator of lipid peroxidation and ferroptosis

Ferroptosis is primarily driven by excessive lipid peroxidation and intracellular iron accumulation. These processes are tightly controlled by the cellular redox environment, which is regulated by an integrated antioxidant network. Among the transcriptional regulators involved, Nrf2 plays a central role in maintaining redox balance, supporting mitochondrial function, and limiting the progression of ferroptosis (44, 45).

Nrf2 modulates several antioxidant and metabolic pathways that counteract the toxic effects of ROS and lipid peroxides. Its activity influences energy metabolism, mitochondrial dynamics, and the transcription of numerous cytoprotective genes. In particular, Nrf2 coordinates the expression of enzymes involved in the biosynthesis and recycling of glutathione (GSH), including glutamate-cysteine ligase catalytic and modifier subunits (GCLC and GCLM), glutathione synthetase (GSS), and the cystine/glutamate antiporter component SLC7A11. In parallel, it regulates the thioredoxin antioxidant system through targets such as thioredoxin-1 (TXN) and thioredoxin reductase-1 (TXNRD1), which collectively maintain the intracellular redox environment (6, 46).

**Figure 2:**
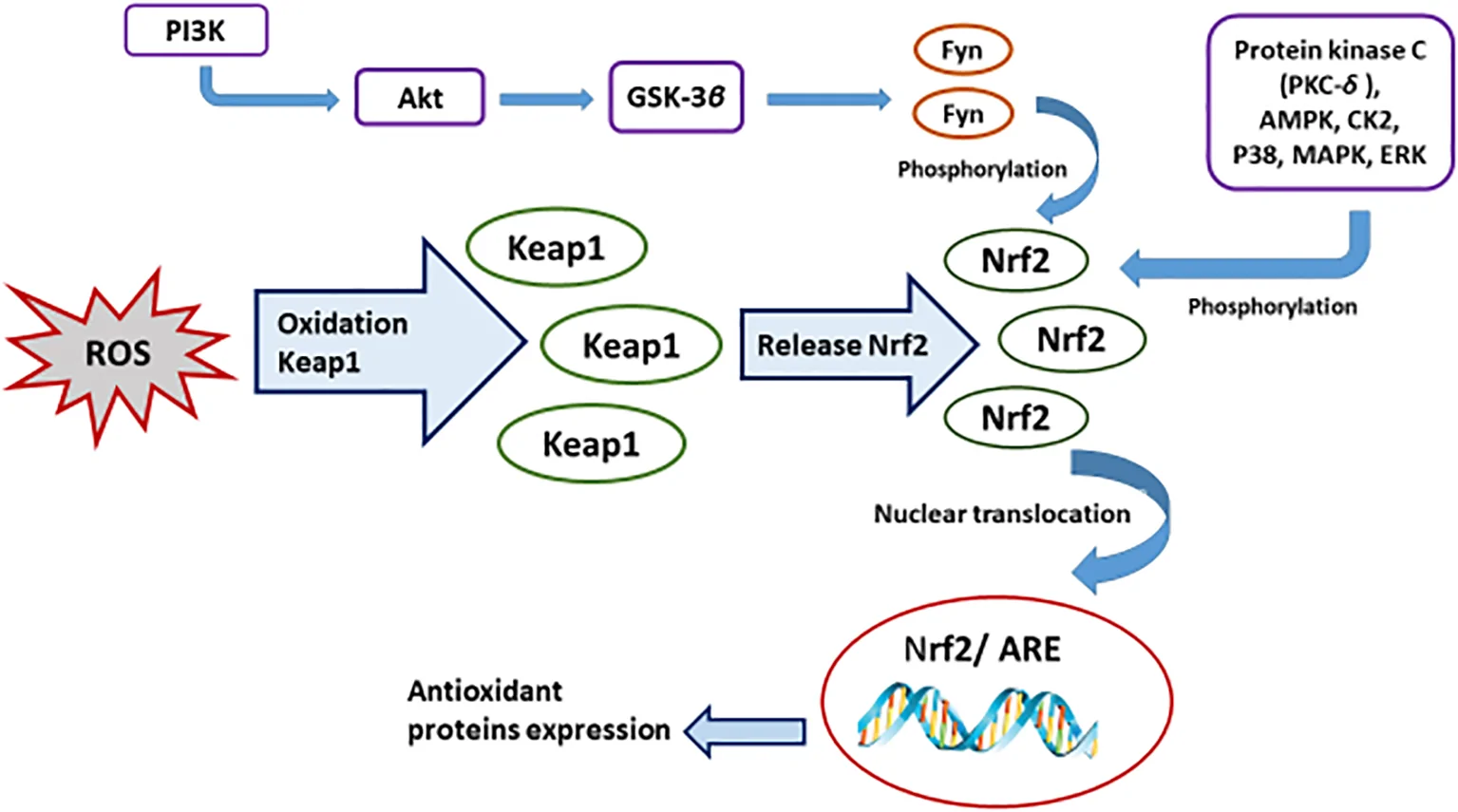
Nrf2/Keap1 pathway under oxidative stress.

The activity of Nrf2 is primarily controlled through its interaction with Kelch-like ECH-associated protein 1 (Keap1), a cytoplasmic repressor that facilitates the ubiquitin-dependent proteasomal degradation of Nrf2 (**Figure 2**).

Since the cloning of Keap1, tremendous progress has been achieved towards comprehending the mechanism of Keap1-mediated negative regulation of Nrf2. It has been postulated that Keap1 works as a molecular switch that is capable to turn the Nrf2-signaling pathway on or off according to intracellular redox circumstances. Serving as a molecular switch, Keap1 exhibits twin functions: it is able to (i) “sense” a disturbance in the redox balance and (ii) turn the Nrf2-mediated response on or off. Recent investigations have examined how these two roles are achieved by Keap1.

Under basal conditions, Keap1 switches the Nrf2-signaling pathway off and maintains low basal levels of Nrf2 by constantly targeting Nrf2 for ubiquitin-mediated protein degradation. When Keap1 “senses” a disturbance in the redox balance, it switches the Nrf2-signaling pathway on.

Under homeostatic conditions, Keap1 binds newly synthesized Nrf2 and targets it for continuous degradation via the proteasome, thereby maintaining low intracellular levels of Nrf2. Upon exposure to oxidative or electrophilic stress, cysteine residues on Keap1 undergo covalent modifications that impair its ability to recognize and ubiquitinate Nrf2. As a result, newly synthesized Nrf2 escapes degradation and translocates into the nucleus, where it binds to antioxidant response elements (AREs) and activates the transcription of a broad array of genes involved in redox regulation, detoxification, and metabolic adaptation (47, 48) (**Figure 2**). Several of the genes regulated by Nrf2 are directly involved in modulating ferroptosis. One of the most important is GPX4, a selenoenzyme that reduces phospholipid hydroperoxides to lipid alcohols. This activity prevents the accumulation of toxic lipid peroxides and preserves membrane integrity, as mentioned above. The function of GPX4 depends on the availability of GSH, whose synthesis and regeneration are under Nrf2 control. In addition, Nrf2 induces the expression of SLC7A11, which promotes cystine uptake and supports GSH production, thereby reinforcing the cellular defense against ferroptosis (49).

Nrf2 also plays a role in iron metabolism, which is closely linked to ferroptotic susceptibility. By promoting the expression of ferritin heavy and light chains (FTH1 and FTL) and the iron exporter ferroportin (FPN1), Nrf2 contributes to the reduction of the labile iron pool, thereby limiting the generation of ROS through the Fenton reaction. In the presence of excess iron, this reaction enhances lipid peroxidation and promotes ferroptotic cell death (50, 51).

When the complex structure of the Keap1-Nrf2 signaling pathway is examined, we see that it is modulated by a system that includes phosphoinositol 3-kinase (PI3K)/protein kinase B (Akt), protein kinase C and mitogen-activated protein kinase molecules. Research showed that several molecules show antioxidant effects through the PI3K/Akt/Nrf2 pathway. The PI3K/Akt signaling pathway regulates Nrf2 activity through GSK-3β, a serine/threonine kinase encoded by the alpha (α) and beta (β) genes. GSK-3β, a key Akt target, has multiple functions, including regulating cell proliferation, apoptosis, glycogen metabolism, and stem cell renewal (52).

The Nrf2-Keap1-ARE signaling axis plays a crucial role in antioxidant defense and is also effective in defense against tumor development. It is also known to play a role in chemotherapeutic drug resistance. Experimental data demonstrate a relationship between impaired autophagy and activation of the Nrf2 pathway. Specifically, p62 facilitates Keap1 degradation via selective autophagy, leading to Nrf2 translocation to the nucleus where it transcriptionally activates downstream antioxidant enzyme expression, protecting cells from OS. Since Nrf2 regulates p62 transcription, a positive feedback loop involving p62, Keap1, and Nrf2 is formed, which further enhances the cell’s protective effects (53).

A controversial issue in recent years is whether cancer cells are dependent on autophagy. One study suggests that while some cells exhibit direct autophagy-dependent development, these cells adapt to bypass the autophagy mechanism. These results demonstrate that resistance to autophagy inhibition is an adaptive mechanism that promotes new tumor formation (54). Studies have shown that ROS-induced autophagy is primarily mediated by O2- during long-term fasting. H_2_O_2_ is produced immediately after fasting and regulates autophagy. Furthermore, the observed relationship between autophagy and ROS varies depending on cell type. For example, autophagy is activated in renal tubular cells as a result of high ROS exposure. Thus, mitochondrial destruction and associated kidney damage can be observed. However, in tumor cells, ROS-induced autophagy activation can promote cell development and cancer progression (55).

The Nrf2-dependent adaptive response, which includes phase II detoxifying enzymes, antioxidants, and transporters that protect cells from further carcinogenic assaults, is how chemopreventive substances or synthetic chemicals carry out their chemopreventive action. As a result, Nrf2 has been considered a “good” protein that shields people from genotoxic harm brought on by carcinogens. Not only does Nrf2 prevent normal cells from becoming cancerous, but it also helps cancer cells survive in harmful environments. Examples of the microenvironmental conditions influencing the dual effects of Nrf2 in cancer include overexpression of the GSTP1 enzyme, Keap1 mutations, loss of heterozygosity, and hypoxia/reoxygenation.

### The role of ferroptosis in cancer biology

Due to their altered redox status and increased iron dependency, cancer cells are particularly susceptible to ferroptosis. This regulated form of cell death, previously discussed in molecular terms, has now been recognized as a crucial determinant in tumor progression and therapy response. Recent studies have highlighted how perturbations in iron metabolism and oxidative stress responses contribute to both the development and suppression of malignancies. While iron accumulation and redox imbalance can promote genomic instability and tumor growth, they also create conditions that sensitize cancer cells to ferroptosis (56).

Inducing ferroptosis has emerged as a promising strategy to overcome resistance to chemotherapy and targeted therapies. Agents such as sorafenib, sulfasalazine, and artesunate have shown efficacy in promoting ferroptotic death in tumor models, particularly in those resistant to apoptosis-based treatments (57). At the same time, defects in ferroptotic signaling and antioxidant systems have been associated with poor prognosis in several cancer types. However, ferroptosis may exert a dual role in cancer, as persistent iron loading, metabolic regulation, and adaptation to oxidative stress may facilitate tumor survival (40, 58, 59).

Emerging evidence also suggests that ferroptosis is tightly coupled to lipid metabolism, membrane repair systems, and oxidative signaling. In tumors with high metabolic plasticity, ferroptosis-targeting strategies must account for the context-specific redox vulnerabilities that define their response to treatment (60, 61).

Nrf2 activation offers extensive and sustained cytoprotection, which is frequently taken over by cancer cells to enable their survival in adverse environments. Furthermore, resistance to chemotherapy, radiation, and immunotherapies is linked to Nrf2 activation in established human malignancies. Tumor-associated macrophages (TAMs) can also activate Nrf2, which promotes an immunosuppressive, anti-inflammatory tumor immune microenvironment (TIME) in addition to cancer cells. Numerous metabolites produced by cancer cells, including itaconate, L-kynurenine, lactic acid, and hyaluronic acid, have been demonstrated to activate Nrf2 and play a significant role in regulating the TIME and tumor-TAMs interaction. Pro-inflammatory cytokines are suppressed, programmed cell death ligand 1 (PD-L1), macrophage colony-stimulating factor (M-CSF), and kynureninase are expressed more frequently, cytotoxic labile heme is catabolized more quickly, and TAMs’ metabolic adaptation is aided. These effects of Nrf2 in TIME are context-dependent and involve several mechanisms. This knowledge offers potential as well as obstacles for the strategic targeting of Nrf2 in cancer (62).

### Pancreatic cancer

Pancreatic ductal adenocarcinoma (PDAC), the most common form of pancreatic cancer, is a highly aggressive malignancy associated with poor prognosis and limited responsiveness to conventional therapies. It frequently originates from oncogenic mutations in the *Kirsten rat sarcoma viral oncogene homolog* (*KRAS*), which are present in over 90% of PDAC cases. Although ferroptosis was initially characterized in the context of *RAS*-driven cell death, it is now established as a distinct form of regulated necrosis initiated by lipid peroxidation. Dysregulation of ferroptosis contributes to both tumor initiation and progression in PDAC. On one hand, ferroptotic damage can promote chronic inflammation through the release of damage-associated molecular patterns (DAMPs), which enhance a tumor-promoting microenvironment. This inflammatory milieu can act synergistically with *KRAS* signaling to facilitate malignant transformation (63, 64).

On the other hand, ferroptosis represents a promising vulnerability in PDAC. The myokine irisin, secreted by skeletal muscle during physical activity, has been proposed as a potential adjunct to ferroptosis-inducing agents. Irisin appears to sensitize PDAC cells to ferroptosis, possibly by modulating redox balance and metabolic pathways (65).

Notably, the impact of ferroptosis in PDAC is modulated by genetic and microenvironmental factors. For example, the mutational status of *tumor protein p53* (*TP53*) influences the cellular response to oxidative damage. Additionally, ferroptosis can promote autophagy and perturb intracellular iron storage, resulting in increased labile iron and further susceptibility to lipid peroxidation. These mechanisms underscore the dual nature of ferroptosis in PDAC, acting as both a suppressive and a tumor-promoting force depending on the context (66).

### Hepatocellular carcinoma

Hepatocellular carcinoma (HCC), the most frequent primary liver malignancy, accounts for over 90% of all liver cancer cases. Its pathogenesis is strongly associated with chronic inflammation, metabolic stress, and iron accumulation—factors that closely intersect with ferroptotic signaling. Recent studies have highlighted the involvement of ferroptosis-related genes in HCC development and therapy response. In particular, resistance to sorafenib, a multi-kinase inhibitor widely used as first-line therapy in advanced HCC, has been linked to impaired ferroptotic mechanisms (67).

Histological analyses of HCC tumors frequently reveal intracellular iron accumulation and elevated lipid peroxide levels (68). Interestingly, some HCC cell lines that are resistant to apoptosis or necrosis remain highly sensitive to ferroptosis-inducing compounds. This suggests that ferroptosis could represent an alternative pathway for eradicating otherwise therapy-resistant tumor cells. Sorafenib has been shown to induce ferroptosis in HCC models by inhibiting the cystine/glutamate antiporter system Xc^-^ and affecting key antioxidant enzymes (69, 70).

### Breast cancer

Breast cancer remains the most commonly diagnosed malignancy worldwide, with its global burden continuing to rise. Accumulating evidence indicates that ferroptosis contributes to both tumor progression and therapeutic resistance in breast cancer, particularly in subtypes lacking effective targeted therapies. Pharmacological induction of ferroptosis has been shown to suppress tumor cell viability, highlighting its potential as a therapeutic avenue (71, 72).

In triple-negative breast cancer (TNBC), one of the most aggressive and treatment-resistant subtypes, ferroptosis appears to be tightly linked to cysteine availability. A study demonstrated that deprivation of cysteine induces ferroptotic death in TNBC cells, which was rescued by deferoxamine (DFO), an iron chelator, and ferrostatin-1, a ferroptosis inhibitor (73). These findings implicate cysteine metabolism as a critical regulator of ferroptosis susceptibility and suggest that metabolic vulnerabilities in TNBC could be exploited to enhance treatment efficacy (74).

### Lung cancer

Lung carcinoma represents a major contributor to global cancer mortality. In experimental models, induction of ferroptosis in lung cancer cells has been associated with cell cycle arrest at the G2/M transition, along with a reduction in the G0/G1 and S phase cell populations. These changes suggest that ferroptotic stress disrupts mitotic progression, impairing the proliferative potential of lung cancer cells (75, 76).

Despite this vulnerability, lung tumors are frequently characterized by resistance to ferroptosis, partly due to their constant exposure to high oxygen levels. Chronic oxidative stress in pulmonary tissue imposes selective pressure for the upregulation of antioxidant systems, allowing tumor cells to counteract ferroptosis-inducing conditions (77). Studies have reported that ferroptosis-related pathways are suppressed in lung cancer, including reduced ROS accumulation and elevated expression of GPX4, a key enzyme involved in lipid peroxide detoxification (78, 79). This adaptive resistance underscores the complexity of ferroptosis regulation in lung tumors and highlights the need for context-specific approaches to restore ferroptotic vulnerability (80, 81).

### Gastric cancer

Gastric cancer (GC) ranks among the most prevalent gastrointestinal malignancies and remains a leading cause of cancer-related deaths worldwide. Current therapeutic strategies rely predominantly on surgical intervention, yet outcomes remain suboptimal in advanced disease stages (82).

Iron metabolism has emerged as a crucial determinant of gastric tumor biology, given the stomach’s central role in systemic iron absorption and regulation. *Helicobacter pylori* infection, a key etiological factor in GC, is strongly associated with iron-deficiency disorders such as anemia (83, 84). Alterations in iron availability not only impact mucosal immunity but may also drive oxidative stress and lipid peroxidation, thereby facilitating malignant transformation. Disrupted iron homeostasis has been shown to influence multiple hallmarks of cancer, including proliferation, angiogenesis, and resistance to cell death, suggesting a mechanistic link between ferroptosis and gastric tumorigenesis (85).

### Nrf2 as a therapeutic target in ferroptosis-driven cancer

Through coordinated regulation of antioxidant capacity, lipid peroxide detoxification, and iron metabolism, the transcription factor Nrf2 emerges as a key modulator of ferroptosis. Under physiological conditions, Nrf2 activation protects cells against oxidative insults by upregulating cytoprotective genes. However, in cancer, sustained activation of Nrf2, often driven by mutations that impair its repressor Keap1, can promote tumor progression, chemoresistance, and escape from ferroptosis. This dual behavior highlights the importance of context when targeting Nrf2 in therapies aimed at modulating ferroptosis (86).

While Nrf2 activation prevents ferroptosis under homeostatic conditions by sustaining antioxidant gene expression, in malignant contexts, it can paradoxically shield tumor cells from ferroptotic stress. Persistent Nrf2 signaling has been associated with enhanced proliferation, metastatic dissemination, and resistance to multiple chemotherapeutic agents. This has positioned the Nrf2 pathway as a major focus in the design of cancer therapies based on ferroptosis induction (87–89).

**Figure 3:**
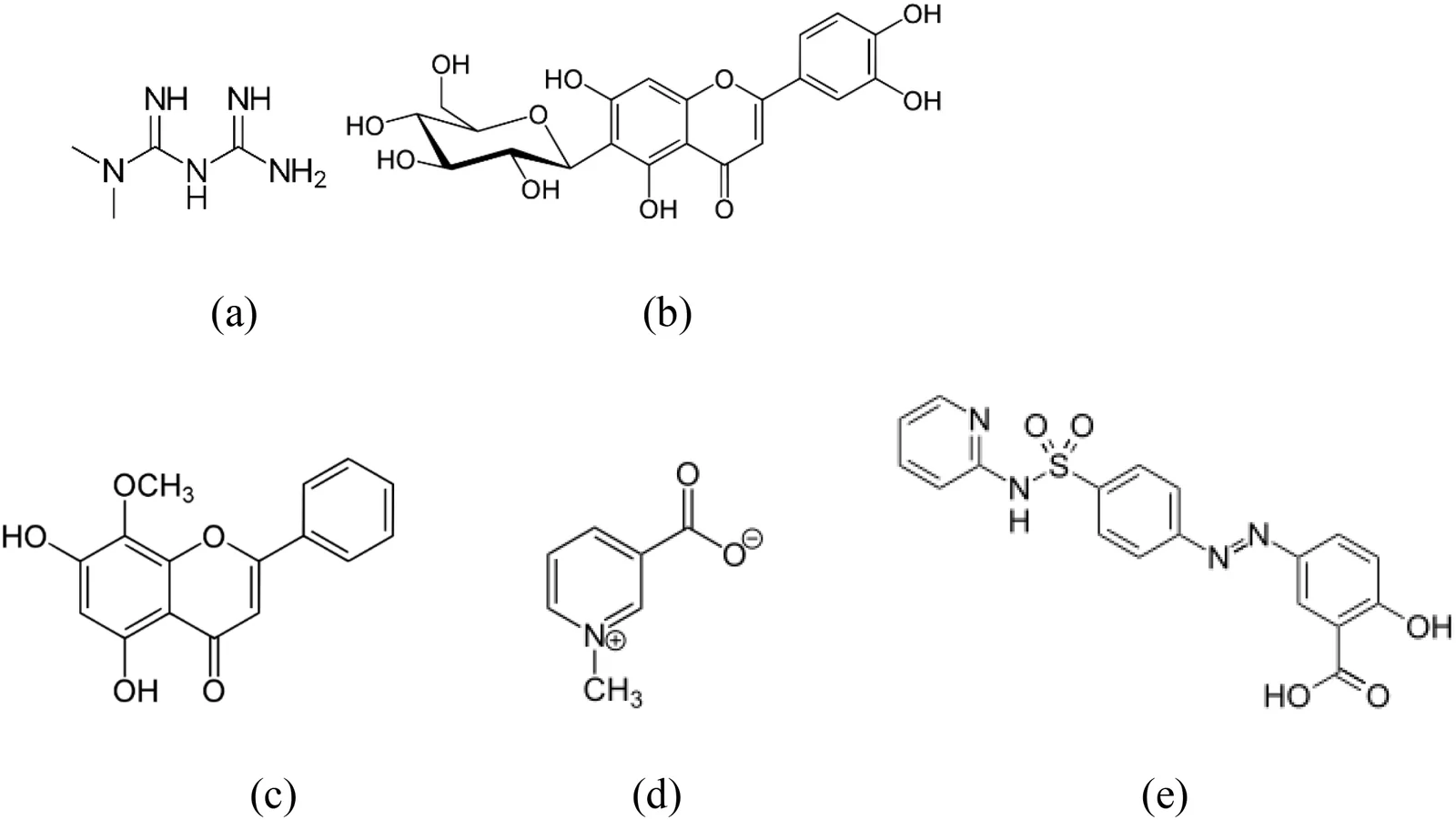
Chemical structures of some Nrf2 inhibitors molecules. metformin **(a)**, isoorientin (homoorientin) **(b)**, wogonin **(c)**, trigonelline **(d)**, sulfasalazine **(e)**.

Multiple pharmacological agents have been identified as modulators of Nrf2 activity. Among the Nrf2 inhibitors, metformin, isoorientin, wogonin, trigonelline, and sulfasalazine have shown antitumor activity by downregulating Nrf2 through diverse mechanisms (**Figure 3**). For example, sulfasalazine inhibit the Nrf2/xCT signaling axis. Metformin impairs Nrf2 nuclear translocation. Isoorientin acts via inhibition of the SIRT6/Nrf2/GPX4 pathway, while trigonelline suppresses Nrf2 through RNA interference (90–94).

**Figure 4:**
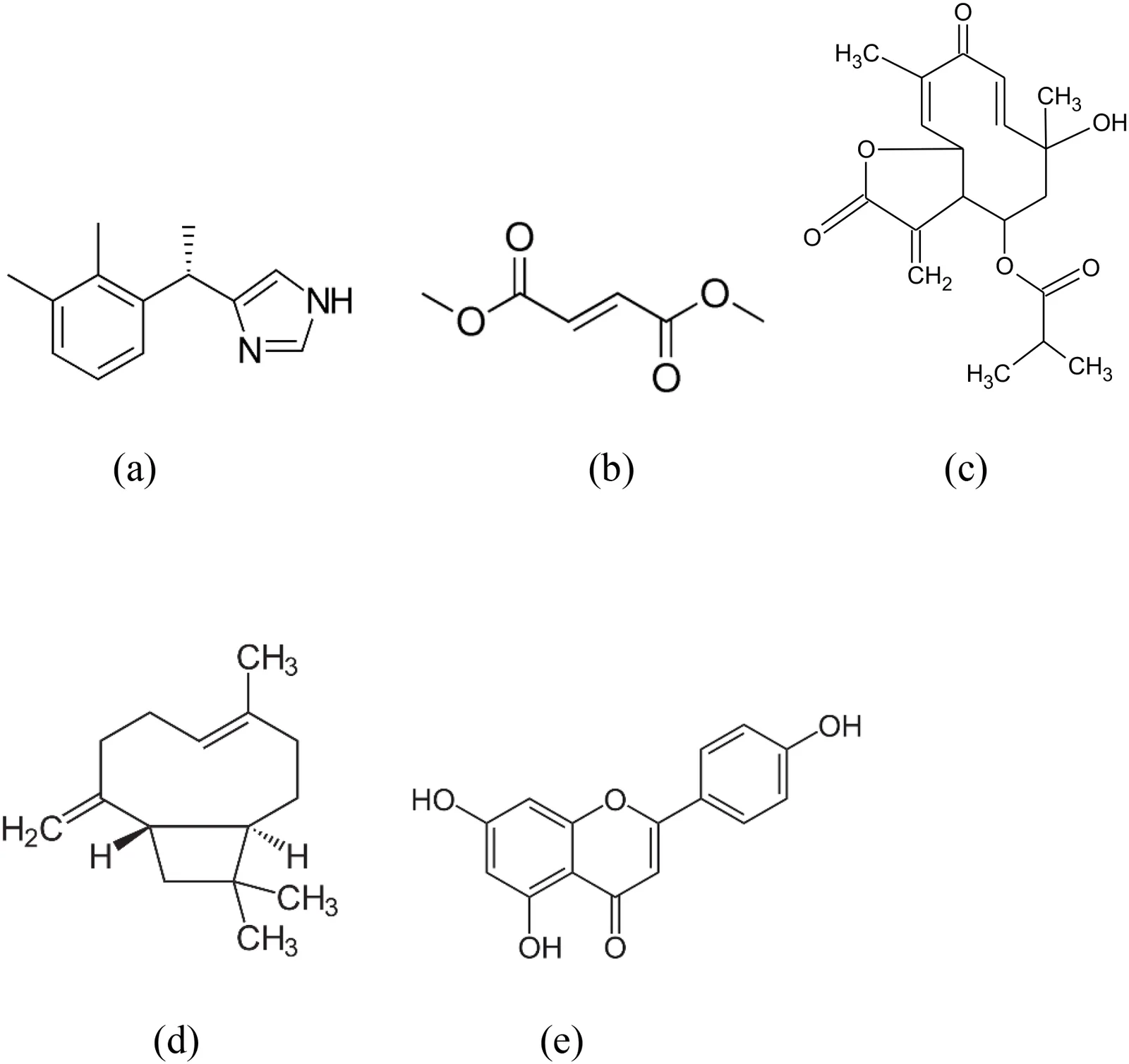
Chemical structures of some Nrf2 activator molecules. Dexmedetomidine **(a)** dimethyl fumarate **(b)**, tagitinin C **(c)**, caryophyllene (BCP) **(d)**, apegenin (APG) **(e)**.

Conversely, several natural and synthetic molecules have demonstrated anticancer properties through activation of Nrf2. These include dexmedetomidine, dimethyl fumarate (DMF), tagitinin C, β-caryophyllene (BCP), and apigenin, as well as molecular regulators such as miR-24, miR-24-3p, Sirt1, and levistilide A. Their mechanisms include activation of the Nrf2/HO-1 pathway (levistilide A, dexmedetomidine), enhancement of Nrf2 nuclear translocation (BCP, Sirt1, tagitinin C, DMF), and stabilization of Nrf2 via inhibition of Keap1 expression (miR-24-3p) (95–103) (**Figure 4**).

This apparent contradiction, whereby both inhibition and activation of Nrf2 are explored as anticancer strategies, reflects the context-dependent nature of Nrf2 signaling. In some tumor types or stages, enhancing Nrf2 activity may restore redox balance, reduce chronic inflammation, and protect normal cells from malignant transformation. In contrast, in established tumors characterized by constitutive Nrf2 activation, further stimulation may exacerbate chemoresistance and tumor growth. This duality underlines the necessity of precise molecular characterization of the tumor microenvironment when considering Nrf2 as a therapeutic target (86).

**Figure 5:**
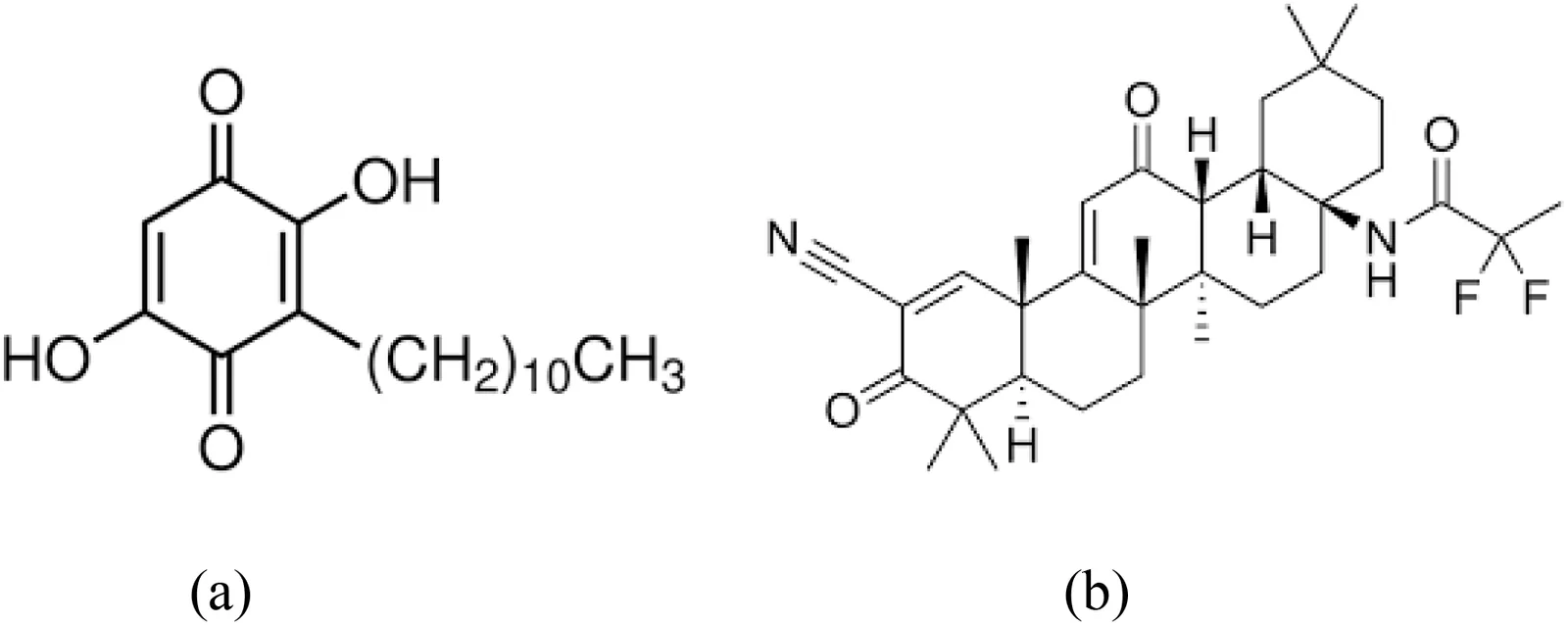
Chemical structures of **(a)** Vatiquinone and **(b)** Omaveloxolone.

Phase 1 clinical trials are ongoing for Nrf2 activators. Phase 3 clinical trials are also underway for vatiquinone, a 15-lipoxygenase inhibitor with both antioxidant and anti-inflammatory effects (104). Vatiquinone (**Figure 5**) target multiple metabolic pathways in the mitochondria. Omaveloxolone (**Figure 5**) is the first FDA-approved drug used against Friedreich’s ataxia (FA). Its mechanism of action is by targeting Nrf2 in the antioxidant pathway (105).

**Figure 6:**
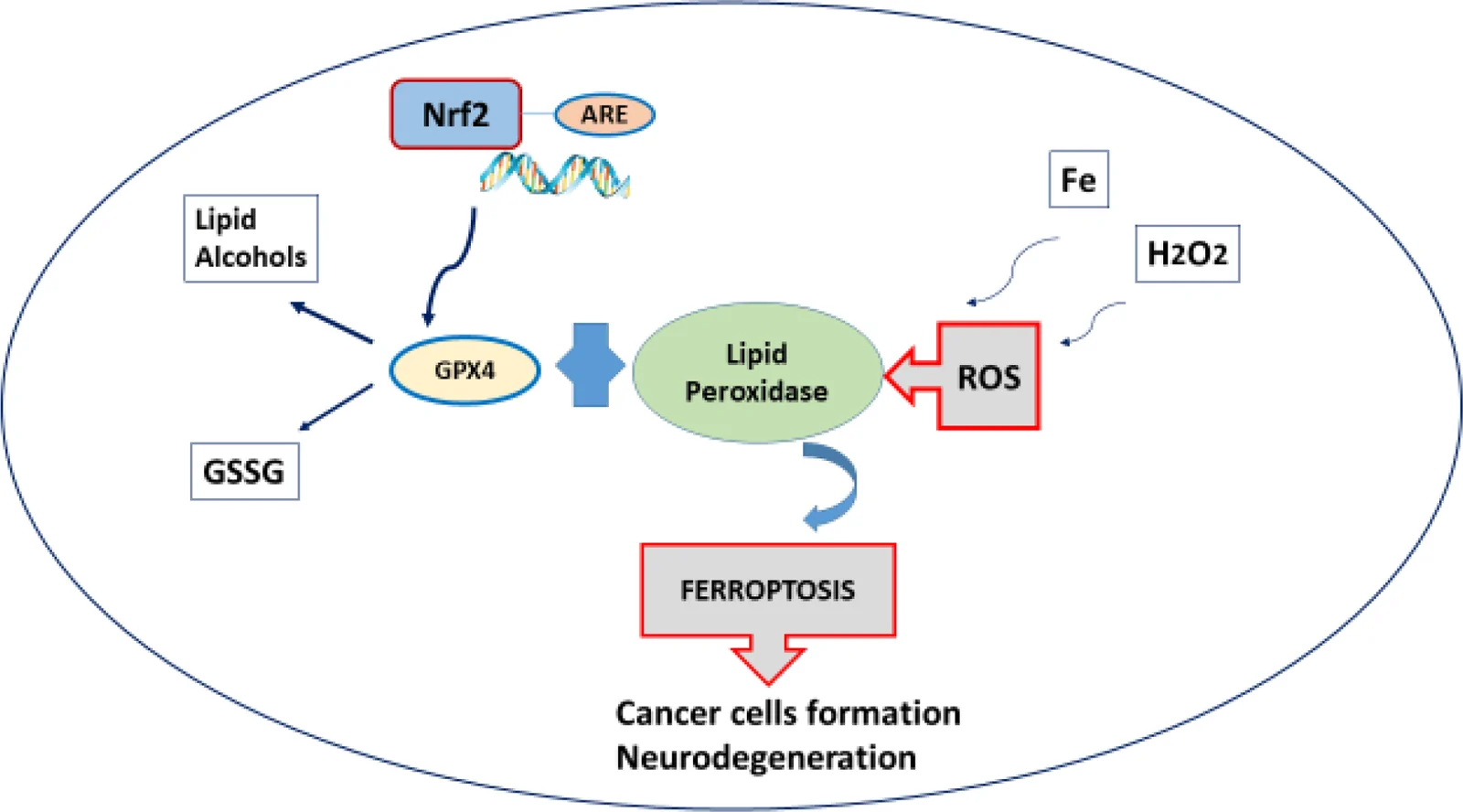
Formation of ferroptosis-related pathologies and the role of Nrf2.

Given its broad regulatory functions, Nrf2 remains a compelling and complex target in ferroptosis-based cancer therapies (**Figure 6**). Its dual roles, protective in normal physiology and potentially oncogenic in malignancy, require precision strategies that take into account the tumor context, mutation profile, and redox environment.

Since Nrf2’s discovery, there has been an increasing amount of evidence demonstrating its beneficial effect in preventing cancer and how it is a crucial transcription factor in shielding people from illnesses linked to oxidative stress. Finding Nrf2 activators for chemoprevention has been the primary focus of research up to this point, but new discoveries indicate that Nrf2 has a “dark” side. According to *in vitro* research, Nrf2 overexpression can raise the expression of a number of intracellular redox-balancing proteins, phase II detoxifying enzymes, and transporters, which can provide cancer cells a growth advantage and result in chemotherapy resistance. It is important to verify the cancer promoting role of Nrf2 observed in cancer cell lines and tumor biopsies, using *in vivo* animal models (106).

Discovering Nrf2’s “dark” side offers a chance for therapeutic intervention against chemoresistance. Chemotherapy with a Nrf2 inhibitor can be utilized to make cancer cells more susceptible to chemical therapy. Nrf2 would be a more effective therapeutic target than a single downstream gene, such HMOX-1 or TrxR, because it is a transcription factor that controls the production of multiple downstream genes that shield cancer cells from apoptosis (106).

## Conclusions

The discovery of ferroptosis as a distinct, iron-dependent form of regulated cell death has significantly advanced our understanding of redox biology and its relevance to cancer progression and therapy. Central to this process is lipid peroxidation, initiated by excessive ROS and altered iron metabolism, which disrupts membrane structure and leads to cell death. Although ROS are integral to physiological signaling, their uncontrolled accumulation shifts the redox equilibrium toward oxidative damage, emphasizing the delicate balance required for cellular homeostasis.

Ferroptosis represents a dual mechanism in oncology. It can suppress tumor growth by eliminating cells with impaired antioxidant capacity, particularly in contexts of chemoresistance or metastasis. However, ferroptotic damage may also promote chronic inflammation and create a tumor-permissive microenvironment, depending on the genetic and immune status of the tissue.

Nrf2 is a critical modulator of this process. Under normal conditions, it preserves redox stability through the regulation of antioxidant and metabolic genes. In cancer, however, its sustained activation, often linked to loss-of-function mutations in Keap1, confers resistance to ferroptosis and facilitates tumor progression. This dual function highlights both the potential and the complexity of targeting Nrf2 in anticancer strategies.

Research on NRF2 cancer still has a lot of unsolved questions. Whether constitutive activation and regulated activation of NRF2 increase the expression of the same target genes is still unknown (107). It wouldn’t be shocking if a particular threshold of NRF2 activation altered the transcriptome, as some research has demonstrated that some target genes are included in the basal but not the induced NRF2 transcriptome and vice versa. Additionally, many potential target genes need to be confirmed, and the NRF2 transcriptome still needs to be completely described. A thorough comprehension of the NRF2 transcriptome will enable us to investigate therapeutic tumor editing and improve our comprehension of the negative aspects of NRF2 in the characteristics of cancer. Furthermore, KEAP1-independent NRF2 control mechanisms should be investigated in relation to cancer treatment and prevention (108). Targeting this transcription factor may be a useful therapeutic strategy, as evidenced by the high frequency of NRF2 in cancer hallmarks. While NRF2 inhibitors may be used to treat cancer, NRF2 activators may be utilized to prevent chemical carcinogenesis (108).

Further investigations should aim to clarify how Nrf2 influences ferroptosis across different tumor contexts, and how its modulation interacts with oncogenic signaling, iron metabolism, and immune regulation. A context-aware approach will be essential to harness ferroptosis as a therapeutic mechanism while minimizing unintended effects on healthy tissues.
