# Correction: Modulating Nrf2 to control lipid peroxidation and ferroptosis: implications for cancer management

**DOI:** 10.3389/fonc.2026.1988777

**Published:** 2026-10-02

**Authors:** Ramazan Tunc, Ilker Ates, Beyza Yılmaz, Alessandro Medoro, Sergio Davinelli, Sibel Suzen, Luciano Saso

**Affiliations:** 1 Department of Pharmaceutical Chemistry, Faculty of Pharmacy, Ankara University, Ankara, Türkiye; 2 Graduate School of Health Sciences, Ankara University, Ankara, Türkiye; 3 Department of Pharmaceutical Toxicology, Faculty of Pharmacy, Ankara University, Ankara, Türkiye; 4 Department of Medicine and Health Sciences “V. Tiberio”, University of Molise, Campobasso, Italy; 5 Department of Physiology and Pharmacology “Vittorio Erspamer”, Sapienza University of Rome, Rome, Italy

**Keywords:** Nrf2, Nrf2 modulators, lipid peroxidation, ferroptosis, oxidative stress, cancer

Affiliation “Graduate School of Health Sciences, Ankara University, Ankara, Türkiye” was omitted for authors “Ramazan Tunc and Beyza Yılmaz”. This affiliation has now been added for authors “Ramazan Tunc and Beyza Yılmaz”.

The original version of this article has been updated.

